# Draft Genome Sequence of Paenibacillus polymyxa 3A-25AI, a Strain Antagonist to Root Rot Causal Phytopathogens

**DOI:** 10.1128/MRA.01464-19

**Published:** 2020-02-13

**Authors:** Jazmín López-Martínez, Yumiko De La Cruz-Rodríguez, Alejandro Alvarado-Gutiérrez, Miguel Alvarado-Rodríguez, Julio Vega-Arreguín, Saúl Fraire-Velázquez

**Affiliations:** a Biología Integrativa de Plantas y Microorganismos, Unidad Académica de Ciencias Biológicas, Universidad Autónoma de Zacatecas, Zacatecas, Mexico; b Unidad Académica de Agronomía, Universidad Autónoma de Zacatecas, Zacatecas, Mexico; c ENES-León, Universidad Nacional Autónoma de México, León, Guanajuato, Mexico; Georgia Institute of Technology

## Abstract

Here, we report the draft genome sequence of the Paenibacillus polymyxa 3A-25AI strain, isolated from the rhizosphere of wild grass. This strain inhibits Phytophthora capsici and Rhizoctonia solani phytopathogens. The genome size is 5.6 Mb, with a G+C content of 45.59%, and contains 5,079 genes, 4,968 coding DNA sequences (CDSs), 35 tRNAs, 3 rRNAs, and 72 unexpected miscellaneous RNA (miscRNA) features.

## ANNOUNCEMENT

Paenibacillus polymyxa is a bacterium that helps to improve biocontrol strategies to counteract phytopathogens. It is a Gram-positive, abundant metabolite and a polysaccharide-producing bacterium (1) that produces nonribosomal peptide/polyketide bioactive compounds with antifungal and antibacterial functions. *P. polymyxa* is also a plant growth-promoting rhizobacterium (2, 3) and improves the microbial richness and diversity of soil (4). A consortium of fungi and oomycete pathogens that cause necrosis commonly affects the roots of solanaceous crops. It is necessary, therefore, to improve phytopathogen biocontrol tools and gradually put aside the use of pesticides.

Here, we report the draft genome sequence of the Paenibacillus polymyxa 3A-25AI strain, isolated from the rhizosphere of Sporobolus airoides (Torr.), sampled in the Morelos Municipality of Zacatecas, Mexico. This bacterial strain grows in King B and LB medium at 27°C, reaching the stationary phase after 18 h.

For sequencing purposes, the bacterium was grown in LB medium for 18 h, and bacterial genomic DNA was extracted with the cetyltrimethylammonium bromide (CTAB) reagent (5). One nanogram of DNA was used for the preparation of libraries by adhering to Nextera kit instructions (Illumina, San Diego, CA, USA). Genome sequencing was performed with a MiSeq sequencer (Illumina) in a 2 × 75-bp paired-end run. The quality of sequencing reads was analyzed in FastQC (6), with a lower threshold for a contig length of 200 bp. Genome assembly was done using SPAdes or Unicycler (7), and the quality of the assemblies was analyzed in QUAST 4.1 (8), including a reference genome (GenBank accession number NZ_CP025957.1). The assembly with SPAdes offered better results, with a contig *N*_50_ value of 16,622 bp and genome coverage of 13.0×. An estimate of the maximum genome completeness and minimal contamination assessed by CheckM (9), including 32 reference genomes and 468 markers, shows 99.09% completeness and 1.25% contamination. Therefore, we have a near-complete sequenced genome with low contamination.

Genome annotation was achieved using the NCBI Prokaryotic Genome Annotation Pipeline (PGAP) (10) and complemented on the Galaxy server using Prokka (11).

**FIG 1:**
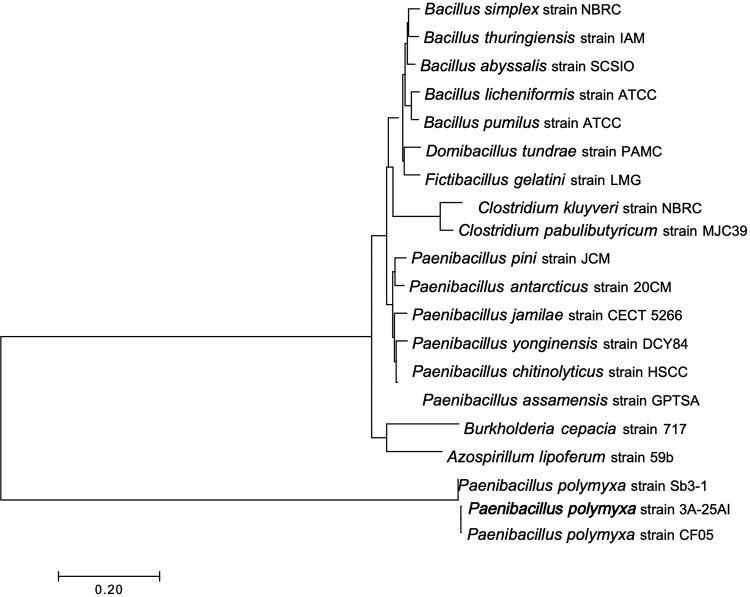
Evolutionary relationships of taxa. The bacterial strain 3A-25AI of this study together with two other *P. polymyxa* strains included in the phylogenetic analysis define a nested clade. The evolutionary history was inferred using the neighbor-joining method (15). The associated taxa clustered together in the bootstrap test (300 replicates). The optimal tree with the sum of branch length of 2.49816535 is shown. The tree is drawn to scale, with branch lengths in the same units as those of the evolutionary distances used to infer the phylogenetic tree. The evolutionary distances were computed using the maximum composite likelihood method and are in the units of the number of base substitutions per site. The analysis involved 20 nucleotide sequences. Codon positions included were 1st + 2nd + 3rd + noncoding. All positions containing gaps and missing data were eliminated. There were a total of 1,246 positions in the final data set. Evolutionary analyses were conducted in MEGA7 (13).

The alignment in NCBI GenBank in the BLAST microbial genomes algorithm unveiled a significant match to Paenibacillus polymyxa SC2, covering 97.5% of contigs, 99.3% of the genome total, and 98.5% identity; the *P. polymyxa* SC2 strain was isolated from the rhizosphere of pepper in Guizhou, China (12). An evolutionary analysis with the 16S rRNA sequence in MEGA7 (13) confirms this bacterium to be Paenibacillus polymyxa (Fig. 1).

The *P. polymyxa* 3A-25AI strain has a genome size of 5.6 Mb; it contains 5,079 genes, 4,968 coding DNA sequences (CDSs), 35 tRNAs, 3 rRNA, and 72 unexpected miscellaneous RNA (miscRNA) features. This bacterial genome has 11 genes devoted to polyketide biosynthesis and 1 β-glucanase. In the analysis using PHASTER (14), 3 prophage regions were identified containing 77 proteins, including integrase, terminase, portal proteins, coat protein, tail shaft, and others. For all software, default parameters were used unless otherwise noted.

The antifungal and antioomycete activities of this bacterium possibly rest on the biosynthesis of polyketides and/or β-glucanase. With the sequenced genome and the described characteristics, this *P. polymyxa* strain adds to the range of possibilities for assembling bacterial formulations for use in the fight against phytopathogens.

### Data availability.

The draft genome sequence of Paenibacillus polymyxa 3A-25AI and the raw data generated in this study have been deposited in DDBJ/ENA/GenBank under the accession number MLCZ00000000. The version described in this paper is the first version, MLCZ01000000. The associated BioProject and BioSample accession numbers are PRJNA343056 and SAMN05846293, respectively.
